# The Association of Hot Red Chili Pepper Consumption and Mortality: A Large Population-Based Cohort Study

**DOI:** 10.1371/journal.pone.0169876

**Published:** 2017-01-09

**Authors:** Mustafa Chopan, Benjamin Littenberg

**Affiliations:** University of Vermont College of Medicine, Burlington, Vermont, United States of America; SERGAS and IDIS, SPAIN

## Abstract

The evidence base for the health effects of spice consumption is insufficient, with only one large population-based study and no reports from Europe or North America. Our objective was to analyze the association between consumption of hot red chili peppers and mortality, using a population-based prospective cohort from the National Health and Nutritional Examination Survey (NHANES) III, a representative sample of US noninstitutionalized adults, in which participants were surveyed from 1988 to 1994. The frequency of hot red chili pepper consumption was measured in 16,179 participants at least 18 years of age. Total and cause-specific mortality were the main outcome measures. During 273,877 person-years of follow-up (median 18.9 years), a total of 4,946 deaths were observed. Total mortality for participants who consumed hot red chili peppers was 21.6% compared to 33.6% for those who did not (absolute risk reduction of 12%; relative risk of 0.64). Adjusted for demographic, lifestyle, and clinical characteristics, the hazard ratio was 0.87 (*P* = 0.01; 95% Confidence Interval 0.77, 0.97). Consumption of hot red chili peppers was associated with a 13% reduction in the instantaneous hazard of death. Similar, but statistically nonsignificant trends were seen for deaths from vascular disease, but not from other causes. In this large population-based prospective study, the consumption of hot red chili pepper was associated with reduced mortality. Hot red chili peppers may be a beneficial component of the diet.

## Introduction

The role of diet in health has become increasingly relevant and investigated. In the past 40 years, the number of PubMed articles indexing diet has increased by at least 700% [1]. While much of the focus has been on macro- and micronutrients, gastronomical aspects have also been investigated.

Peppers and other spices have long been used to color, flavor and preserve foods, as well as for medicinal purposes. Based on the theories of Hippocrates and Galen, spices were thought to help restore the humoral imbalances responsible for disease and illness in medieval times [2]. The benefits of spices and their bioactive compounds have since been suggested by various *in vitro*, *in vivo* and experimental models. Pungent spices, for example chili peppers, increase lipid catabolism in different organs and tissues [3–5], which could protect against hypercholesterolemia and obesity, reducing the risks of hypertension, type 2 diabetes, and atherosclerotic cardiovascular disease. The anti-microbial activity of spices, as highlighted by inhibitory effects against *H*. *pylori* and other bacteria and fungi [6–8], may alter the gut microbiota and influence various metabolic diseases [9–11]. Many spices possess antioxidant [12] and anti-inflammatory effects [13–15], and could serve to prevent and mitigate various chronic diseases.

The potential anti-tumorigenic properties [16] of some spices have been supported by an ecological study of the U.S. and India, which revealed an inverse relationship between spice production (and, presumably, consumption) and cancer incidence [17]. A prospective cohort analysis in China provided the most convincing evidence to date of the clinical benefits of spices, particularly peppers [18]. It showed an inverse relationship between chili pepper consumption and mortality from all-causes, cancer, respiratory and cardiovascular disease.

Although data on the consumption patterns in various populations are not available, other groups may differ significantly in the amount and type of peppers used compared to the Chinese. The association between peppers and health has not been studied on a large scale in the west. We sought to measure the association between hot red chili pepper consumption and mortality in a large and representative population of US adults.

## Materials and Methods

We studied the National Health and Nutritional Examination Survey (NHANES), a representative sample of the US noninstitutionalized population. Data were analyzed from a subsample of participants in the NHANES III survey conducted from 1988 to 1994. We included all subjects at least 18 years old with complete data for the outcome and the predictors. NHANES participants undergo extensive interviews and laboratory assessments including blood and urine tests and measures of socioeconomic factors, clinical characteristics and personal habits [19]. Usual consumption of foods and drinks during the past month was assessed with an 81-item food frequency questionnaire administered at baseline; however, portion size was not assessed. Vital status provided by the National Death Index was linked with the dataset through December 31, 2011. NHANES was originally a cross-sectional survey; however, the inclusion of follow-up vital status enables a prospective cohort analysis.

The primary predictor was hot red chili pepper consumption per month, derived from subjects’ responses to “How often did you have hot red chili peppers? Do not count ground red chili peppers.” The primary outcome was long-term mortality. Chili pepper consumers were classified as subjects who reported any value greater than zero when asked about the frequency of hot red chili pepper intake per month.

Deaths were ascertained by a probabilistic matching algorithm with the National Death Index, based on various identifying information (social security number, first and last name, date of birth, *etc*.), or death certificate when possible [20]. Causes of death were classified as Heart Disease (ICD-10 codes I00-I09, I11, I13, I20-I51), Cancer (C00-C97), Chronic Pulmonary Diseases (J40-J47), Accidents (V01-X59, Y85-Y86), Stroke (I60-I69), Dementia (G30), Diabetes (E10-E14), Pneumonia including influenza (J09-J18), Kidney Disease (N00-N07, N17-N19, N25-N27) and all other causes. We also created a combined category of all Vascular Disease including deaths from either Heart Disease or Stroke.

The personal and laboratory characteristics considered as covariates were chosen to minimize the possibility of confounding by social characteristics and personal habits that were plausibly associated with either hot red chili pepper consumption or mortality. Age, gender, education, race, ethnicity, marital status, employment, annual income, physical activity, and consumption of meats, vegetables and fruits were reported by the participants. Educational attainment was divided into four categories: none, grade school, middle school, or high school graduate or beyond. Race was categorized as White, Black, or Other. Ethnicity was categorized as Mexican-American, other Hispanic, or non-Hispanic. Low income was defined as annual income less than $20,000 at the time of the interview in 1984–1994. Marital status was defined as married, divorced or separated, widowed, or never married. Frequency of fruit intake per month included a summative value of numerical responses for orange juice, other fruit juices, citrus fruits, melons, peaches, nectarines, and any other fruits. Similarly, vegetable intake per month included carrots, broccoli, Brussel sprouts/cauliflower, spinach, greens, tossed salad, cabbage, coleslaw, sauerkraut, and any other vegetables. Meat intake per month included bacon, sausage, processed meats, liver and other organ meats, beef, pork and ham. Current use of cigarettes was defined as a confirmatory response to “Do you now smoke cigarettes?” Current use of alcohol was defined as reporting using alcohol in the last 12 months. Because the duration of various exercises were not solicited at the time of survey, physical activity level was indirectly measured, as outlined in a prior study [21], and subjects were classified into three categories: no regular exercise, regular low-to-moderate exercise, or regular vigorous exercise.

Additional characteristics of the subjects representing the possible intermediate effects of hot red chili pepper consumption were also collected. Body measurements were taken by trained NHANES staff using standard protocols [22]. Body Mass Index (BMI) was calculated by dividing weight in kilograms by height in meters squared. Obesity was specified as BMI ≥ 30 kg/m^2^. Cholesterol measurements were standardized according to criteria from the CDC and Prevention Lipid Standardization Program [23, 24]. HDL and total cholesterol values are based on serum samples taken regardless of fasting state. The presence of diabetes was determined by a glycated hemoglobin A1C level ≥6.5% or fasting serum glucose > 125 mg/dl or positive response to “Have you ever been told by a doctor that you have diabetes or sugar diabetes?” The presence of hypertension was determined by a systolic blood pressure ≥ 140 mmHg or diastolic blood pressure ≥ 90 mm Hg or a positive response to both “Because of your (high blood pressure/hypertension), have you ever been told by a doctor or other health professional to take prescribed medicine?” and “Are you now taking prescribed medicine?”

## Analysis

Data were retrieved from the NHANES website [19] and statistical analyses were conducted with Stata 14.1 (StataCorp, College Station, Texas). Wilcoxon-rank sum and chi-squared tests were used to compare cohort characteristics for continuous and categorical variables, respectively. Association between the predictor and outcome was assessed by multivariate Cox proportional hazards regressions.

Three models were devised. Model 1 consisted of the predictor (hot red chili pepper consumption) and the outcome (mortality). Model 2 added demographic and socioeconomic information to the prior model. Personal habits were added for Model 3. Because markers of obesity, hypertension, diabetes or inflammation might be intermediate factors in the potential causal pathway between hot red chili pepper consumption and mortality, we did not include them in the multivariate models. We calculated hazard ratios and their 95% confidence intervals (CI). A two-tailed *P* <0.05 was required for statistical significance. We also calculated the unadjusted absolute risk reduction associated with hot red chili pepper consumption and it’s inverse, the number of subjects who would need to consume hot red chili peppers to prevent one death (analogous to the number-needed-to-treat [25]). All analyses were adjusted for the stratified sampling scheme used in NHANES to represent the non-institutionalized US adult population [26].

We performed secondary analyses of the relationship of hot pepper consumption and cause-specific mortality using Cox proportional hazards models adjusted for the same variables retained in Model 3 above.

## Results

**Fig 1:**
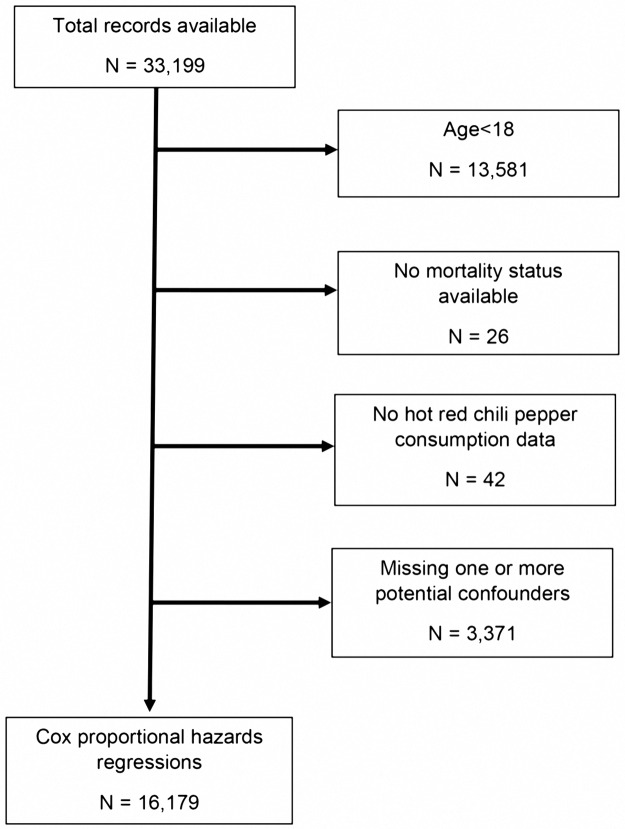
Subject Inclusion.

The NHANES III survey for 1988 to 1994 included 33,199 records. After excluding children and adults missing either the outcome, the main predictor, or any of the covariates, 16,179 eligible participants were analyzed (Fig 1).

Table 1 presents the baseline characteristics of the participants according to hot red chili pepper consumption. Compared with participants who did not consume hot red chili peppers, those who did consume them were more likely to be younger, male, white, Mexican-American, married, and to smoke cigarettes, drink alcohol, and consume more vegetables and meats. They had lower HDL-cholesterol, lower income, and less education.

**Table 1:** Characteristics of the study population.

*Characteristic*	Does not consume chili peppers			Consumes chili peppers			P*
	N = 12,071			N = 4,107			
	*Mean or percent*	*Median*	*Range*	*Mean or percent*	*Median*	*Range*	
Age (years)	48.2	45	18–90	41.9	38	18–90	<0.001
Male sex	43.0%			58.1%			<0.001
Race							<0.001
White	65.4%			76.0%			
Black	32.2%			18.9%			
Other	2.5%			5.2%			
Ethnicity							<0.001
Mexican-American	16.6%			58.3%			
Other Hispanic	2.8%			1.6%			
Non-Hispanic	80.6%			40.1%			
Marital status							<0.001
Married	58.0%			64.8%			
Divorced or separated	11.2%			10.2%			
Never married	19.2%			19.6%			
Widowed	11.6%			5.4%			
Education							<0.001
None	7.4%			17.4%			
Grade School	12.4%			16.0%			
Middle School	50.6%			42.6%			
High School graduate	29.6%			24.0%			
Annual income < $20,000	47.0%			54.0%			<0.001
Current smoker	24.9%			28.0%			0.001
Current drinker	42.2%			54.7%			<0.001
Physical activity							<0.001
No regular activity	26.8%			30.2%			
Regular low-to-moderate	64.7%			60.6%			
Regular vigorous	8.5%			9.2%			
Diet (portions per month)							
Fruits	49	42	0–619	50	41	0–622	0.19
Vegetables	51	44	0–374	55	49	0–309	<0.001
Meats	23	19	0–624	25	21	0–156	<0.001
Obesity	25.4%			23.9%			0.02
Diabetes	12.1%			10.4%			0.011
Hypertension	27.1%			19.9%			<0.001
Total cholesterol (mg/dl)	205	201	59–702	202	199	81–491	0.003
HDL cholesterol (mg/dl)	51	49	12–196	50	48	8–158	<0.001
Years of observation	17	19	0–23	18	19	0–23	<0.001

Continuous variables were compared using Wilcoxon’s rank-sum test. Categorical variables were compared with the chi-square test.

During a median follow-up of 18.9 years (range 1 day-23 years) including 273,877 person-years of observation), we observed 4,946 deaths. Unadjusted mortality for participants who consumed hot red chili peppers was 21.6% (887/4,107) compared to 33.6% (4.059/12,071) for those who did not, resulting in an absolute risk reduction of 12% (*P*<0.001; 95% CI -13.5, -10.5) and a relative risk of 0.64. The number-needed-to-prevent one death was 8.3.

In model 1, the unadjusted Cox-proportional hazard ratio was 0.59 (*P*<0.001; CI 0.52, 0.68). See Table 2. With the addition of socioeconomic and demographic variables (model 2), the hazard ratio became 0.87 (*P* = 0.02, CI 0.78, 0.98). The inclusion of a diet and lifestyle habits (model 3) had little impact with a hazard ratio of 0.87 (*P* = 0.01; CI 0.77, 0.97).

**Table 2:** Cox proportional hazards models of the effect of eating hot peppers on total mortality among 16,179 adults in NHANES III.

*Variable*	Model 1			Model 2			Model 3		
	*HR*	*CI*	*P*	*HR*	*CI*	*P*	*HR*	*CI*	*P*
**Eats hot peppers**	**0.59**	**0.52, 0.68**	**<0.001**	**0.87**	**0.77, 0.98**	**0.020**	**0.87**	**0.77, 0.97**	**0.014**
Age at Interview				1.09	1.09, 1.10	<0.001	1.10	1.09, 1.10	<0.001
Sex				1.62	1.50, 1.75	<0.001	1.65	1.50, 1.80	<0.001
Race = White				1.00	-	-	1.00	-	-
Black				1.17	1.04, 1.31	0.01	1.09	0.98, 1.21	0.11
Other				0.72	0.49, 1.06	0.09	0.66	0.44, 0.97	0.034
Ethnicity = Mexican-American				1.00	-	-	1.00	-	-
Other Hispanic				0.68	0.56, 0.84	<0.001	0.64	0.51, 0.81	<0.001
Non-Hispanic				1.14	0.998, 1.30	0.053	1.12	0.99, 1.28	0.08
Education = None				1.00	-	-	1.00	-	-
Grade School				0.93	0.78, 1.12	0.47	0.97	0.80, 1.18	0.75
Middle School				0.97	0.82, 1.15	0.73	1.02	0.85, 1.22	0.85
High School Grad				0.71	0.59, 0.86	0.001	0.83	0.68, 1.01	0.064
Marital Status = Married				1.00	-	-	1.00	-	-
Divorced/Separated				1.25	1.06, 1.48	0.01	1.21	1.02, 1.44	0.031
Never married				1.24	1.003, 1.53	0.047	1.29	1.05, 1.59	0.017
Widowed				1.03	0.93, 1.15	0.53	1.006	0.90, 1.12	0.90
Income < $20K				1.57	1.43, 1.72	<0.001	1.48	1.35, 1.62	<0.001
Current Smoker							1.87	1.66, 2.11	<0.001
Current Drinker							0.89	0.80, 0.98	0.26
Fruits per month							0.999	0.999, 1.0001	0.98
Vegetables per month							1.0006	0.999, 1.002	0.31
Meat portions per month							1.001	0.9997, 1.003	0.13
Activity Level = No Regular Exercise							1.00	-	-
Regular Low-to-moderate							0.73	0.65, 0.81	<0.001
Regular Vigorous							0.72	0.57, 0.89	0.004

4,946 deaths over 23 years of observation; HR = hazard ratio; CI = 95% confidence interval

Analyses of specific causes of death (Table 3), adjusted for all the variables in Model 3 above, showed similar (but statistically nonsignificant) magnitudes of reduction in the hazard of death from heart disease and stroke, but not from other causes.

**Table 3:** Cox proportional hazards models of the effect of eating hot peppers on cause specific mortality among 16,179 adults in NHANES III.

*Cause of Death*	*Number of deaths*	*HR*	95% CI	*P*
**Total**	**4,946**	**0.87**	**0.77, 0.97**	**0.01**
Heart Disease	1,211	0.81	0.61, 1.07	0.14
Cancer	1,069	0.89	0.70, 1.13	0.33
Chronic Pulmonary Disease	216	1.03	0.64, 1.67	0.89
Accidents	141	1.14	0.55, 2.36	0.73
Stroke	352	0.64	0.35, 1.16	0.14
Dementia	103	0.88	0.30, 2.54	0.80
Diabetes	178	0.76	0.37, 1.55	0.45
Pneumonia or Influenza	147	0.81	0.35, 1.92	0.63
Kidney Disease	74	1.30	0.36, 4.68	0.68
Other	1,401	0.87	0.67, 1.15	0.32
Vascular disease (Heart disease or Stroke)	1,563	0.86	0.74, 1.001	0.052

All models are adjusted for age, sex, race, ethnicity, education, marital status, income, consumption of alcohol, fruits, vegetables and meats, and physical activity at baseline. HR = hazard ratio; CI = 95% confidence interval.

## Discussion

In this large prospective study, we observed an inverse relationship between hot red chili pepper consumption and all-cause mortality, after adjusting for potential confounders. Adults who consumed hot red chili peppers had a 13% lower hazard of death, compared to those who did not. These results add to the literature by corroborating the main results of an earlier study [18]. They are distinct in that they are drawn from a different population and thus support the generalizability of the protective effects of hot red chili peppers.

Although the mechanism by which peppers could delay mortality is far from certain, Transient Receptor Potential (TRP) channels, which are primary receptors for pungent agents such as capsaicin (the principal component in chili peppers), may in part be responsible for the observed relationship. Activation of TRP vanilloid type 1 (TRPV1) appears to stimulate cellular mechanisms against obesity, by altering mediators of lipid catabolism and thermogenesis [27]. Protection against obesity leads to decreased risk of cardiovascular, metabolic and lung diseases. Capsaicin may also defend against heart disease via a TRP-mediated modulation of coronary blood flow [28]. Capsaicin’s antimicrobial properties [29] may indirectly affect the host by altering the gut microbiota. For instance, changes in bacterial composition, production of metabolites, and number of colonies have been linked to obesity [30], diabetes [9], cardiovascular disease [10] and liver cirrhosis [11], although the mechanisms for these associations are unknown. Nuclear factor kappa-light-chain-enhancer of activated B cells (NF-κB), an important regulator of cellular growth, is inactivated by various spices, including capsaicin, and could mediate anti-tumor effects [31]. Finally, hot red chili peppers also contain other nutrients, including B-vitamins, vitamin C and pro-A vitamin, which may partly account for its protective effect.

This analysis has several important limitations. Although the narrow confidence intervals and small *P*-values argue against random error, the apparent association between hot red chili pepper consumption and mortality could be due to confounding by some factor not controlled in the analysis. For example, differences in other foods often consumed with hot red chili peppers, such as other spices, could serve as potential confounders. In fact, one or more of the social or demographic variables included in Model 2 appear to partially confound the unadjusted association between red hot chili pepper consumption and mortality, as demonstrated by the change in Hazard Ratio between Model 1 and Model 2. Nonetheless, the association remains statistically and epidemiologically significant, with no evidence of further confounding by the additional lifestyle characteristics included in Model 3.

Dietary information was gathered in a cross-sectional manner, and may not represent long-term consumption of hot red chili peppers. “Hot red chili peppers” could include a variety of different types, and may represent a narrower selection than seen in the Chinese study (perhaps because of the qualifier “red”). With the exception of ground peppers, the data do not allow for delineation between fresh or dried peppers. The limitations of dietary recall are well recognized [32]. Nonetheless, dietary intake data in the NHANES survey are collected using standardized, validated protocols [33, 34].

The sample cohort is representative of a U.S. non-institutionalized population, but not necessarily generalizable to other groups. The NHANES III survey was conducted during the years 1988 to 1994, and recent patterns of chili pepper consumption may differ.

Cause-specific mortality analysis in this data set are limited by the relatively small numbers of deaths. Nonetheless, the trends towards protection from deaths due to vascular disease stands in contrast to the lack of any apparent protection from death due to accidents, chronic pulmonary disease and kidney disease, and may provide a clue to hot red chili pepper’s mechanism of action.

Our analyses showed a significant decrease in mortality associated with hot red chili pepper consumption. The results support the findings of Lv, *et al*. [18]. which revealed an inverse relationship between spicy food consumption and mortality, and strengthen its generalizability. Given the observational nature of both investigations, causality can only be suggested, not confirmed. Further studies should aim to investigate the benefits of other spices and differential effects of certain chili pepper subtypes. Such evidence may lead to new insights into the relationships between diet and health, updated dietary recommendations, and the development of new therapies.
